# Auto-GNN: Neural architecture search of graph neural networks

**DOI:** 10.3389/fdata.2022.1029307

**Published:** 2022-11-17

**Authors:** Kaixiong Zhou, Xiao Huang, Qingquan Song, Rui Chen, Xia Hu

**Affiliations:** ^1^DATA Lab, Department of Computer Science, Rice University, Houston, TX, United States; ^2^Department of Computing, The Hong Kong Polytechnic University, Kowloon, Hong Kong SAR, China; ^3^LinkedIn, Sunnyvale, CA, United States; ^4^Samsung Research America, Silicon Valley, CA, United States

**Keywords:** graph neural networks, automated machine learning, neural architecture search, deep and scalable graph analysis, reinforcement learning

## Abstract

Graph neural networks (GNNs) have been widely used in various graph analysis tasks. As the graph characteristics vary significantly in real-world systems, given a specific scenario, the architecture parameters need to be tuned carefully to identify a suitable GNN. Neural architecture search (NAS) has shown its potential in discovering the effective architectures for the learning tasks in image and language modeling. However, the existing NAS algorithms cannot be applied efficiently to GNN search problem because of two facts. First, the large-step exploration in the traditional controller fails to learn the sensitive performance variations with slight architecture modifications in GNNs. Second, the search space is composed of heterogeneous GNNs, which prevents the direct adoption of parameter sharing among them to accelerate the search progress. To tackle the challenges, we propose an automated graph neural networks (AGNN) framework, which aims to find the optimal GNN architecture efficiently. Specifically, a reinforced conservative controller is designed to explore the architecture space with small steps. To accelerate the validation, a novel constrained parameter sharing strategy is presented to regularize the weight transferring among GNNs. It avoids training from scratch and saves the computation time. Experimental results on the benchmark datasets demonstrate that the architecture identified by AGNN achieves the best performance and search efficiency, comparing with existing human-invented models and the traditional search methods.

## 1. Introduction

Graph neural networks (GNNs) (Micheli, 2009) have emerged as predominant tools to model graph data at various domains, such as social media (Grover and Leskovec, 2016) and bioinformatics (Zitnik and Leskovec, 2017). Following the message passing strategy (Hamilton et al., 2017), GNNs learn a node's representation *via* recursively aggregating the representations of its neighbors and itself. The learned node representations could be employed to deal with different tasks efficiently.

The success of GNNs is usually accompanied with careful architecture parameter tuning, aiming to adapt GNNs to the different types of graph data. For example, attention heads in the graph attention networks (Velickovic et al., 2017) are selected for the citation networks and the protein–protein intermodule data. These human-invented architectures not only require the manual trials in selecting the architecture parameters, but also tend to obtain the suboptimal performance when they are transferred to other graph data. Based on these observations, we investigate how to automatically identify the optimal architectures for the different scenarios.

Neural architecture search (NAS) has attracted increasing research interests (Elsken et al., 2018). Its goal is to find the optimal neural architecture in the predefined search space to maximize the model performance on a given task. It has been widely reported that the new architectures discovered by NAS algorithms outperformed the human-invented ones at many domains, such as the image classification (Zoph and Le, 2016) and semantic image segmentation (Liu et al., 2019). Motivated by the previous superior success of NAS, we propose to investigate whether an efficient and effective NAS framework could be developed for the network analytics problems.

However, the direct application of existing NAS algorithms to find GNN architectures is non-trivial, due to the two challenges as follows. *First, the traditional search controller of NAS is inefficient to discover a well-performing GNN architecture*. GNNs are specified by a sequence of modules, including aggregation, combination and activation. Considering node classification task, the classification performances of GNNs vary significantly with the slight modification of a module. For example, over graph convolutional networks (GCN) (Kipf and Welling, 2017), the test accuracy drops even if we slightly change the aggregation function from sum to mean pooling. The traditional controller samples the whole module sequence to formulate a new architecture at each search step. After validating the new architecture, the controller gets updates as the result of the mixed module modifications. It would be hard for the traditional controller to learn the following relationship: which part of the architecture modifications improves or degrades the model performance. *Second, the widely adopted technique in NAS such as parameter sharing (Pham et al.*, *2018**) is not suitable to GNN architectures*. The parameter sharing trains common weights and transfers them to every newly sampled architecture, aiming to avoid training from scratch and measure the new architecture quickly. But it fails to share weights between any two heterogeneous GNN architectures, which have the distinct output statistics. The output statistics of a model is defined by the mean, variance, or value interval of its neuron activation values. Suppose that we have weights deeply trained in a GNN architecture with Sigmoid activation function, bounding the neural outputs within interval [0, 1]. If we transfer the weights to another architecture possessing Linear function with loose activation interval [−∞, +∞], the neural output values may be too large to be back propagated steadily by the gradient decent optimizer.

We propose the automated graph neural networks (AGNN) to tackle the aforementioned challenges. Specifically, it could be separated as answering two research questions. (i) How do we design the search controller tailored to explore the well-performing GNN architectures efficiently? (ii) Given the emerging heterogeneous GNN architectures during the search progress, how do we make the parameter sharing feasible? In summary, our contributions are described as follows:

- We build up the most comprehensive search space to cover the *elementary, deep* and *scalable* GNN architectures. The search space incorporates the recent techniques, such as skip connections and batch training, to explore the promising models on large-scale graphs.
- We design an *efficient* controller by considering the key property of GNN architectures into search progress—the variation of node distinguishing power with slight architecture modifications.
- We define the heterogeneous GNN architectures in the context of parameter sharing. A constrained parameter sharing strategy is proposed to enhance the functional *effectiveness* of transferred weights in the new architecture.
- We conduct the extensive experiments to search the elementary, deep, and scalable GNNs, which delivers the most superior results on both small and large-scale graph datasets. Comparing with existing NAS, AGNN achieves the double wins in the search efficiency and effectiveness.

## 2. Related work

### 2.1. Graph neural networks

The core idea of GNNs is to learn the node embedding representations recursively from the representations at the previous layer. The graph convolutions at each layer is realized by a series of manipulations, including the message passing and self updating. A variety of GNNs based on spatial graph convolutions has been developed, including GNN models with the different aggregation mechanisms (Hamilton et al., 2017; Corso et al., 2020), and the different attentions (Vaswani et al., 2017; Velickovic et al., 2017). Recently, the deep GNNs have been widely studied to learn the high-order neighborhood structures of nodes (Chen et al., 2020; Zhou et al., 2020). Given the large-scale graphs in real-world application, several scalable GNNs are proposed by applying the batch training (Chang and Lin, 2010; Zeng et al., 2019).

### 2.2. Neural architecture search

NAS has been widely explored to facilitate the automation of designing and selecting good neural architectures. Most of NAS frameworks are built up based on reinforcement learning (RL) (Baker et al., 2016; Zoph and Le, 2016). RL-based approaches adopt a recurrent controller to generate the variable-length strings of neural architectures. The controller is updated with policy gradient after evaluating the sampled architecture on the validation set. To tackle the time cost bottleneck of NAS, parameter sharing (Pham et al., 2018) is proposed to transfer the weights well trained before to a new sampled architecture, and avoids training from scratch.

### 2.3. Graph NAS

As far as we know, the only prior work on conjoining the researches of GNNs and NAS is GraphNAS (Gao et al., 2019). To be specific, GraphNAS directly applies the reinforcement learning search method and the traditional parameter sharing. Following this pioneer work, the recent efforts of graph NAS either modify the search space for their specific downstream tasks (Ding et al., 2020; You et al., 2020; Zhao et al., 2020a; Cai et al., 2021; Wei et al., 2021), or apply the different search methods (Li and King, 2020; Shi et al., 2020; Zhao et al., 2020b). For example, targeting at the graph classification problem, previous work (Cai et al., 2021; Wei et al., 2021) incorporates the operation of feature filtration or graph pooling into the search space. Besides the reinforcement learning searched algorithm, several differentiable search frameworks have been developed to improve search efficiency. For example, Zhao et al. (2020b) and Ding et al. (2021) relax the discrete search space to be continuous, where each graph module is represented by the probabilistic combination of various candidate functions. The evolutionary algorithm is used to generate architecture with generic operations of crossover and mutation (Shi et al., 2020).

## 3. Search space

Before going to the technique details, we first unify the terminologies used under the graph NAS framework. We use the term “architecture” to refer to an available graph neural networks that could be applied for the downstream application. Specifically, GNN architecture is characterized by multiple independent dimensions, such as aggregation function and hidden units. Along each architecture dimension, there are a series of candidate modules provided to support the automated architecture engineering. For example, we have candidates {SUM, MEAN, MAX} at the dimension of aggregation function. The search space is then constructed by Cartesian product of all the dimensions, which contains a large amount of available architectures. NAS is to iteratively sample the next architecture, and moves toward the optimal architecture in the search space as close as possible (Chen et al., 2021).

Following the popular message passing strategy (Gilmer et al., 2017), GNNs are stacked by a series of graph convoutional layers. Formally, the graph convolutions at the *k*-th layer are:


(1) $$\begin{array}{l} h_{i}^{\left(k\right)}=\text{AGGRE}(\{a_{ij}^{\left(k\right)}W^{\left(k\right)}x_{j}^{\left(k-1\right)}:j\in N(i)\});\\ x_{i}^{\left(k\right)}=\sigma (\text{COM}(W^{\left(k\right)}x_{i}^{\left(k-1\right)},h_{i}^{\left(k\right)})). \end{array}$$

$x_{i}^{\left(k\right)}\in {\mathbb{R}}^{d^{\left(k\right)}}$ denotes the representation embedding of node *i* learned at the *k*-th layer. N(i) denotes the set of neighbors adjacent to node *i*. *W*^(*k*)^∈ℝ^*d*^^(*k*)^×*d*^(*k*−1)^ is trainable weight. $a_{ij}^{\left(k\right)}$ denotes the edge weight between nodes *i* and *j*. Functions AGGRE and COM are applied to aggregate neighbor embeddings and combine them with the node itself, respectively. σ denotes the activation function. Based on above equation, we define the comprehensive search space to support the searches of elementary, deep and scalable GNNs for various applications. We categorize the search dimensions as following, and list their candidate modules in Supplementary Section S2.

- **Elementary dimensions**. We use the term “elementary GNNs” to represent the widely applied models in literature, which often contain less than three layers. The elementary dimensions are: (**I**) hidden units specifying *d*^(*k*)^; (**II**) attention function used to compute $a_{ij}^{\left(k\right)}$; (**III**) number of attention heads; (**IV**) aggregation function; (**V**) combination function; and (**VI**) activation function.
- **Deep dimensions**. We include dimension of (**VII**) skip connections to allow the stacking of deep GNNs. To be specific, at layer *k*, the embeddings of up to *k* − 1 previous layers could be sampled and combined to the current layer's output.
- **Scalable dimensions**. The dimension of (**VIII**) batch size is included to facilitate the computation on large-scale graphs.

We highlight that most of existing search space only cover the elementary dimensions (Gao et al., 2019). In particular, although the batch size is contained in the search space of You et al. (2020), it is used for the graph classification instead of the node classification problem concerned in this work. The technical implementation of batch sampling for these two problems are significantly different: While the graph classification samples independent graphs as a batch similar to tradition machine learning tasks, the node classification samples dependent nodes to formulate a subgraph. We are aware that the skip connections are searched in Zhao et al. (2020a). But it only optimize the connection choice at the last layer of a three-layer GNN. They fail to explore the deep and scalable models (Chiang et al., 2019; Chen et al., 2020), which have been recently widely explored to boost GNNs' performances.

## 4. Reinforced conservative controller

In the traditional search controller of reinforcement learning (RL)-based NAS, a recurrent neural networks (RNN) encoder is applied to specify the neural architecture strings (Zoph and Le, 2016; Pham et al., 2018). At each search step, the RNN encoder will sample the string elements one by one, and use them to formulate a new architecture. After validating the new architecture, a scalar reward is used to update the RNN encoder. However, it is problematic to directly apply this traditional controller to find the well-performing GNN architectures. The main reason is that GNNs' performances may vary significantly with the slight modifications along a single dimension (e.g., aggregation function). The traditional controller would be hard to learn about which part of architecture modifications contributing more or less to the performance improvement, thus failing to identify the powerful modules of a certain dimension in the future search process.

**Figure 1:**
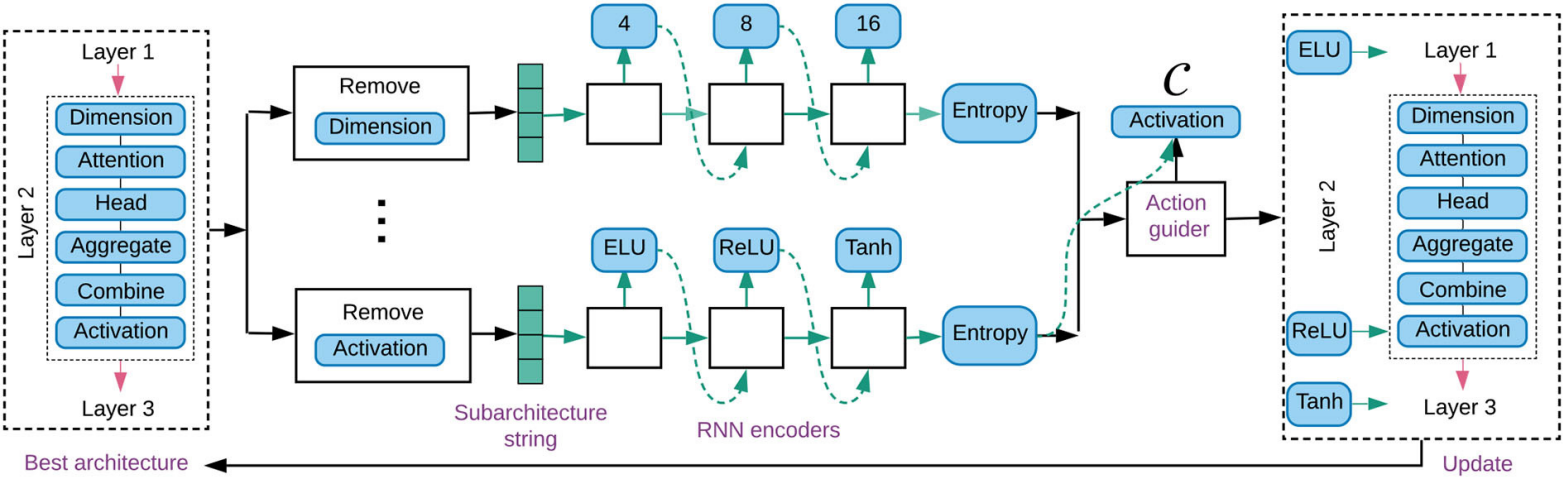
Illustration of AGNN with a three-layer GNN search along elementary dimensions. Controller takes the best architecture as input, and applies RNN encoder to sample the alternative modules for each dimension. We select the dimension (e.g., activation function) deserved to be explored, and modify the preserved best architecture with the alternative modules.

In order to search GNN architectures efficiently, we propose a new controller named reinforced conservative neural architecture search (RCNAS), as shown in Figure 1. It is built up upon RL-based exploration boosted with conservative exploitation. To be specific, there are three key components: (1) a conservative exploiter, which screens out the best architecture found so far; (2) a guided architecture explorer, which slightly modifies the modules of certain dimensions in the preserved best architecture; and (3) a reinforcement learning trainer that learns the relationship between the slight architecture modifications and model performance change.

### 4.1. Conservative exploiter

The conservative exploiter is applied to keep the best architecture found so far. In this way, the following architecture modifications are performed upon a reliable parent architecture, which ensures fast exploitation toward the better offspring architectures in the huge search space. If the offspring GNN outperforms its parent, the best neural architecture is updated; otherwise, it will be kept and reused to generate the next offspring GNN.

### 4.2. Guided architecture explorer

The guided architecture explorer is proposed to modify the best architecture, *via* choosing the dimensions deserved for exploration. As shown in Figure 1, we use the example of the activation function being selected. Correspondingly, the modules of activation function in a three-layer GNN architecture are changed to ELU, ReLU, and Tanh, respectively. The details are introduced as follows.

#### 4.2.1. RNN encoders

As shown in the middle part of Figure 1, for each dimension *c*, an RNN encoder is implemented to sample a series of new modules. These modules are potential to be used to update the *n* layers in the preserved GNN correspondingly. First, a subarchitecture string is generated by removing the original modules of the concerned dimension. This subarchitecture represents the input status that asks for module padding. Second, following an embedding layer, the *n* new modules are sampled layer by layer.

Specifically, RNN encoder of dimension *c* decides the sampling probability distribution at layer *k* as: ${\text{P}}_{k}^{c}=P\left(k|\theta^{c}\right)\in {\mathbb{R}}^{m\times 1},1\leq k\leq n$. θ^*c*^ denotes the trainable parameters, and *m* denotes the module cardinality. The module at layer *k* is randomly sampled based on distribution ${\text{P}}_{k}^{c}$. Reusing the example of activation function dimension in Figure 1, {ELU, ReLU, Tanh} are generated and prepared to modify the preserved architecture.

#### 4.2.2. Modification guider

It is responsible to choose the architecture dimensions to modify the preserved GNNs. We note that NAS is encouraged to explore the search space along the direction with a great amount of uncertainty. The uncertainty of a dimension could be defined by the entropy of sampling probability. Formally, the decision entropy of dimension *c* is: $E_{c}=\overset{n}{\underset{i=1}{\sum }}-{\text{P}}_{i}^{c}\log{\text{P}}_{i}^{c}$. The larger the *E*_*c*_ is, the higher is the probability of exploring uncertain dimension *c*.

Given the decision entropies {⋯ , *E*_*c*_, ⋯ } of all the dimensions, the modification guider randomly chooses dimensions $C=\left\{c_{1},\cdots \;,c_{s}\right\}$ with size |C|=s. We use the default value of *s* = 1 to achieve the goal of minimum architecture modification. We provide the hyperparameter study of *s* in Appendix, which shows the model performance generally decreases with the increasing of size *s*. This validate our motivation to explore the search space of GNN architectures with small steps.

#### 4.2.3. Architecture modifier

We modify the modules of the preserved best architecture according to list C. For each dimension within list C, the corresponding original modules are replaced with the newly sampled ones. Considering the case of C={activation function} in Figure 1, the sampled modules {ELU, ReLU, Tanh} are applied for the activation functions in the three-layer GNN, while keeping the other modules in the preserved architecture unchanged. After the architecture modifications, the offspring GNN is evaluated to estimate its model performance.

### 4.3. Reinforcement learning trainer

We use REINFORCE rule (Sutton et al., 2000) to update RNN encoder. For each modified dimension c∈C, we compute the gradients of parameters θ_*c*_ by the following rule (Zoph and Le, 2016):


(2) $$\begin{array}{l} \nabla_{\theta_{c}}J\left(\theta_{c}\right)=\overset{n}{\underset{k=1}{\sum }}𝔼\left[\left(R-B_{c}\right)\nabla_{\theta_{c}}\log{\hat{P}}_{k}^{c}\right]. \end{array}$$

${\hat{P}}_{k}^{c}$ represents the probability of sampled module at layer *k*, given by the corresponding element from vector ${\text{P}}_{i}^{c}$. *R* denotes the reward (i.e., validation performance) by evaluating the new offspring architecture. *B*_*c*_ denotes the reward baseline of dimension *c* for variance reduction in reinforcement learning. Let *M*_*b*_ and *M*_*o*_ denote the model performances of the preserved best architecture and the new offspring, respectively. We propose the following reward shaping: *R* = *M*_*o*_ − *M*_*b*_, which represents the model performance variation due to the architecture modification. Using the same reward, RNN encoders of all the *s* dimensions within list C are updated based on Eq. (2).

The proposed RCNAS solves the inefficiency problem in the conventional controller by utilizing a small value of *s*. The conventional controller used in GraphNAS (Gao et al., 2019) generates modules of all the dimensions to formulate a new architecture each time, which is mathematically equivalent to *s*≫1 in RCNAS. Reward *R* is obtained as the result of mixed architecture modifications on all the dimensions. When updating a specific RNN encoder, REINFORCE rule will introduce noise derived from the other dimensions. It is hard to distinguish the contribution of module samples of each dimension to the final model performance. RNN encoder will fail to learn the following relationship accurately: the model performance and the module selections of certain dimension. In contrast, by applying the extreme case of *s* = 1 in RCNAS, reward *R* is estimated by slightly modifying one architecture dimension. REINFORCE rule only updates the corresponding RNN encoder to learn the above relationship exclusively. This would facilitate the controller to identify the powerful modules of each dimension, and explore the well-performing offspring architectures.

## 5. Constrained parameter sharing

Compared with training from scratch, the parameter sharing reduces the computation cost by forcing the explored neural architecture to share the common weights (Pham et al., 2018). The transferred weights should work effectively in the new architecture, and estimate its performance as accurately as training from scratch. However, the traditional strategy cannot share weights among the heterogeneous GNN architectures stably for a few reasons. We say that two neural architectures are heterogeneous if they have the significantly distinct output statistics. For example, the output intervals of activation functions Sigmoid and Linear are [0, 1] and [−∞, +∞], respectively. The activation values of Linear may be overly large in the offspring architecture, if its weights are transferred from ancestor equipped with activation function of Sigmoid. The output explosion would lead to unstable training of the offspring architecture. Furthermore, the trainable weights in connection layers, like batch normalization and skip connections, are deeply coupled in the ancestor architecture to connect the specific successive layers. These weights are hard to be transferred to the offspring to bridge another successive layers well.

**Figure 2:**
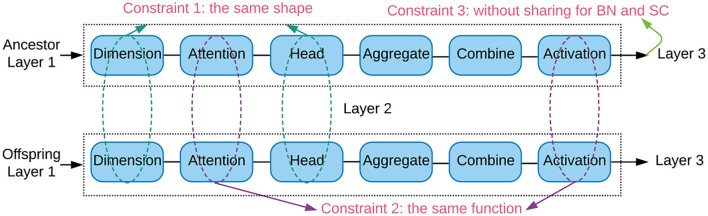
Illustration of the constrained parameter sharing strategy between the ancestor and offspring architectures in layer 2. The trainable weights of a layer are shared when they have the same shapes (constraint 1), attention and activation functions (constraint 2). Constraint 3 avoids sharing in the batch normalization (BN) and skip connection (SC).

To tackle the above challenges, we propose constrained parameter sharing strategy as illustrated in Figure 2. The trainable weights are transferred in a layer-wise fashion. For each layer in the new offspring architecture, it share weights from suitable ancestor by satisfying three constraints:

- The ancestor and offspring architectures have the same shapes of trainable weights, in order to enable the transferred weights being used directly. The weight shapes are specified by both the hidden units and attention heads.
- The ancestor and offspring architectures have the same attention and activation functions. The attention function collects the relevant neighbors, and the activation function squashes the output to a specific interval. Both of them largely determine the output statistics of a layer.
- The weights of the connection layers are not shared. The connection layers contain the batch normalization and skip connections. We train each offspring architecture with a few epochs (e.g., 5 or 20 epochs in our experiment) to adapt these connection weights to the new successive layers.

## 6. Experiments

We experiment on the node classification task with the goal of answering the five research questions. **Q1:** How does the elementary GNN architecture discovered by AGNN compare with the human-invented models and the ones searched by other methods? **Q2:** How effective is AGNN to build up the deep architecture? **Q3:** How scalable is AGNN to explore superior model on large-scale graphs? **Q4:** How efficient is the proposed RCNAS controller compared with the ones in other search methods? **Q5:** Whether the constrained parameter sharing transfers weights effectively to the offspring architectures? We provide the explanation of discovered architectures in Supplementary Sections S4–S6.

### 6.1. Datasets

To study the neural architecture search of elementary and deep GNNs, we use the benchmark node classification datasets of Cora, Citeseer, and Pubmed (Sen et al., 2008) under the transductive setting, and apply PPI under the inductive setting (Zitnik and Leskovec, 2017). To search the scalable GNNs, we use large-scale graphs of Reddit (Hamilton et al., 2017) and ogbn-products (Hu et al., 2020). Their dataset statistics are in Supplementary Section S1.

### 6.2. Baseline methods

- **Human-invented GNNs** : The message-passing based GNNs as shown in Eq. (1) are considered for fair comparison, except the one combined with the pooling layer or other advanced techniques. Considering the elementary GNNs, we apply baseline models of Chebyshev (Defferrard et al., 2016), GCN (Kipf and Welling, 2017), GraphSAGE (Hamilton et al., 2017), GAT (Velickovic et al., 2017), LGCN (Gao et al., 2018). For deep GNNs, we consider state-of-the-art (SOTA) models of PairNorm (Zhao and Akoglu, 2019), SGC (Wu et al., 2019), JKNet (Xu et al., 2018), and APPNP (Klicpera et al., 2018). For scalable GNNs, we use baseline models of GraphSAGE (Hamilton et al., 2017), Cluster-GCN (Chiang et al., 2019), and GraphSAINT (Zeng et al., 2019).
- **NAS approaches:** We note that most of existing NAS methods cannot be applied directly to search deep and scalable GNNs. We use GraphNAS (Gao et al., 2019), the most popular NAS model based on reinforcement learning, as baseline to search elementary GNNs. The random search is implemented to sample architectures randomly, serving as a strong baseline to evaluate the efficiency and effectiveness of the sophisticated NAS.

### 6.3. Training details

Following the previous configurations (Velickovic et al., 2017; Gao et al., 2018), we search the two-layer and three-layer elementary GNNs for the transductive and inductive learning, respectively. The layer numbers for deep model search and scalable model search are 16 and 3, respectively. A total of 1,000 architectures are explored iteratively during the search progress. The classification accuracies are averaged *via* randomly initializing the optimal architecture 10 times. The details of training hyperparameter setting are listed in Supplementary Section S3.

### 6.4. Results

#### 6.4.1. Search of elementary GNNs

We search the elementary GNNs with 2–3 layers, and compare with the human-invented GNNs and NAS methods to answer question **Q1**. The test performances of human-invented GNNs are reported directly from their papers. Tables 1, 2 summarize the classification results and parameter sizes for the transductive and inductive learning, respectively. We make the following observations.

**Table 1:** Classification accuracy (in percent) under transductive learning.

**Framework**	**Model**		**Cora**		**Citeseer**		**Pubmed**	
		**#Layers**	**#Params**	**Accuracy**	**#Params**	**Accuracy**	**#Params**	**Accuracy**
GNNs	Chebyshev	2	0.09M	81.2	0.09M	69.8	0.09M	74.4
	GCN	2	0.02M	81.5	0.05M	70.3	0.02M	79.0
	GAT	2	0.09M	83.0 ± 0.7	0.23M	72.5 ± 0.7	0.03M	79.0 ± 0.3
	LGCN	2	0.05M	81.6 ± 0.4	0.12M	70.4 ± 1.1	0.02M	77.3 ± 1.2
NAS	GraphNAS-w/o share	2	0.09M	82.7 ± 0.4	0.23M	73.5 ± 1.0	0.03M	78.8 ± 0.5
	GraphNAS-with share	2	0.07M	83.3 ± 0.6	1.91M	72.4 ± 1.3	0.07M	78.1 ± 0.8
	Random-w/o share	2	0.37M	81.4 ± 1.1	0.95M	72.9 ± 0.2	0.13M	77.9 ± 0.5
	Random-with share	2	2.95M	82.3 ± 0.5	0.95M	69.9 ± 1.7	0.13M	77.9 ± 0.4
AGNN	AGNN-w/o share	2	0.05M	**83.6** **±0.3**	0.71M	**73.8** **±0.7**	0.07M	**79.7** **±0.4**
	AGNN-with share	2	0.37M	82.7 ± 0.6	1.90M	72.7 ± 0.4	0.03M	79.0 ± 0.5

Datasets are based on full splitting: all nodes except those in the validation and test sets will be used for training. The bold values indicate the highest accuracy at each column.

# Number of parameters.

**Table 2:** Test accuracy of the human-invented and searched architectures under the inductive learning.

**Framework**	**Model**	**Layers**	**PPI**	
			**Params**	**F1 score**
GNNs	GraphSAGE	2	0.39M	0.612
	GAT	3	0.89M	0.973 ± 0.002
	LGCN	4	0.85M	0.772 ± 0.002
	GraphNAS-w/o share	3	4.1M	0.985 ± 0.004
NAS	GraphNAS-with share	3	1.4M	0.960 ± 0.036
	Random-w/o share	3	1.4M	0.984 ± 0.004
	Random-with share	3	1.4M	0.977 ± 0.011
AGNN	AGNN-w/o share	3	4.6M	**0.992** **±0.001**
	AGNN-with share	3	1.6M	0.991 ± 0.001

The bold values indicate the highest accuracy at each column.

❶ *The neural architectures discovered by AGNN without parameter sharing achieve the most superior accuracies on all the benchmarks*. Comparing with the human-invented GNNs, AGNN without parameter sharing delivers the average improvement of 2.8%, owing to the careful selection of each architecture axis. Comparing with GraphNAS and random search, our AGNN is more effective to explore the outperforming models. At each search step, the whole neural architecture is sampled and reconstructed in GraphNAS and random search. In contrast, our AGNN exploits the best architecture to provide a reliable start, and explores the search space by only modifying a specific module class. Therefore, it provides a good trade-off between the exploitation and exploration to pursue the outperforming models.

❷ *AGNN with parameter sharing generally outperforms the human-invented GNNs*. Although the parameter sharing brings performance deterioration, they could accelerate the search progress by avoiding training from scratch for each searched models. We provide trade-off study between the model performance and computation time in the following.

#### 6.4.2. Search of deep GNNs

To answer question **Q2**, we search the deep GNNs with 16 layers, and compare with other search algorithms as well as SOTA models. We also include the evolutionary algorithm as an another strong baseline, where the best architecture found so is reserved for the following random mutation.

We note that GraphNAS cannot be directly applied to search deep model due to its simplified search space. The classification accuracies are listed (Table 3). ❸ *The results show that the novel deep architectures identified by AGNN consistently deliver the outperforming accuracies*. Comparing with the human-invented models, AGNN optimizes the skip connections at each layer to tackle the over-smoothing issue, which is the key bottleneck in developing deep GNNs (Zhou et al., 2020). Due to the large search space of skip connections, the random search may be inefficient to explore the well-performing architectures given the certain search steps.

**Table 3:** Classification accuracy (in percent) of 16-layer GNNs.

**Model**	**Cora**	**Citeseer**	**Pubmed**
GCN	22.02 ± 6.24	19.78 ± 1.95	37.94 ± 0.53
PairNorm	44.23 ± 7.26	27.45 ± 7.22	68.59 ± 7.30
SGC	72.10 ± 0.00	71.03 ± 1.18	70.20 ± 0.00
JKNet	74.54 ± 3.72	54.33 ± 7.74	69.98 ± 6.26
APPNP	79.38 ± 0.62	72.13 ± 0.53	77.07 ± 0.66
Random	83.76 ± 0.42	71.55 ± 0.94	79.01 ± 0.47
AGNN	**84.06** **±0.29**	**72.04** **±0.89**	**79.51** **±0.32**

Datasets are based on public splitting. The bold values indicate the highest accuracy at each column.

#### 6.4.3. Search of scalable GNNs

To answer question **Q3**, we further search the training batch size of GNNs on large-scale graphs: Reddit and ogbn-products. We note that all of other NAS frameworks cannot support the scalable optimization on graphs with more than 200K nodes. Comparing with scalable GNNs and random search, we list the classification accuracy in Table 4. ❹ *By searching the appropriate batch size, skip connections, etc., AGNN could explore the outperforming scalable GNNs for each large-scale dataset*.

**Table 4:** Node classification accuracies (in percent) on large-scale graphs.

**Model**	**Reddit**	**ogbn-products**
GraphSASE	95.96 ± 0.03	78.70 ± 0.36
ClusterGCN	95.94 ± 0.05	78.97 ± 0.33
GrarphSAINT	95.46 ± 0.08	79.08 ± 0.24
Random	95.90 ± 0.04	79.13 ± 0.58
AGNN	**96.47** **±0.04**	**79.37** **±0.69**

The bold values indicate the highest accuracy at each column.

#### 6.4.4. Search efficiency comparison

**Figure 3:**
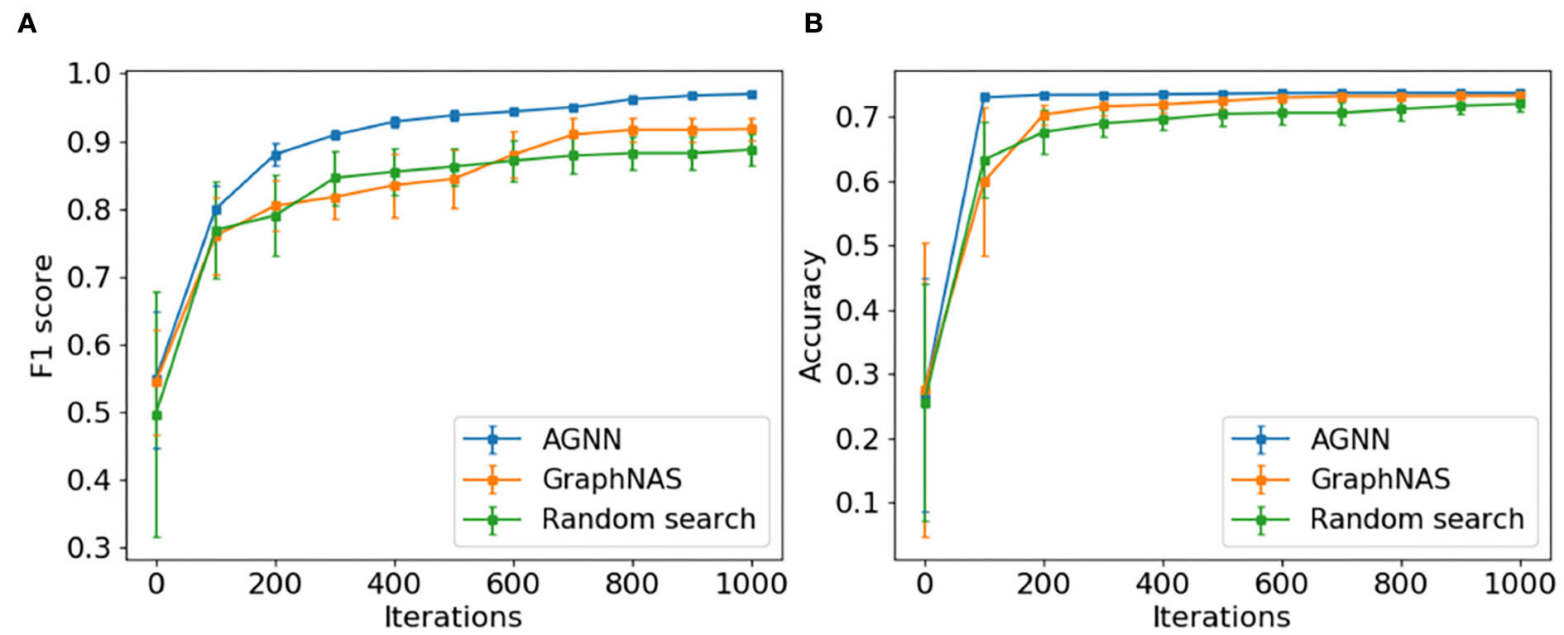
The progression of top-10 averaged performance of AGNN, GraphNAS, and random search. **(A)** PPI and **(B)** Citeseer.

To answer question **Q4**, we compare the search efficiencies of AGNN, GraphNAS and random search. Given the total of 1,000 search steps, the search efficiency is represented by the progression of the average performance in the top-10 architectures found so far. From Figure 3, we make the following observation.

❺ *AGNN is much faster to identify the well-performing architectures during the search progress*. At each step, the top-10 architectures discovered by AGNN have the better average performance comparing with GraphNAS and random search. The remarkable efficiency of AGNN is contributed by the exploitation and conservative exploration abilities within RCNAS controller: the best architecture is used preserved for modification; and the smallest amendment on one module class is applied. As explained before, RCNAS controller optimizes the module samples accurately to push the search direction toward the good architectures.

#### 6.4.5. Parameter sharing effectiveness

**Figure 4:**
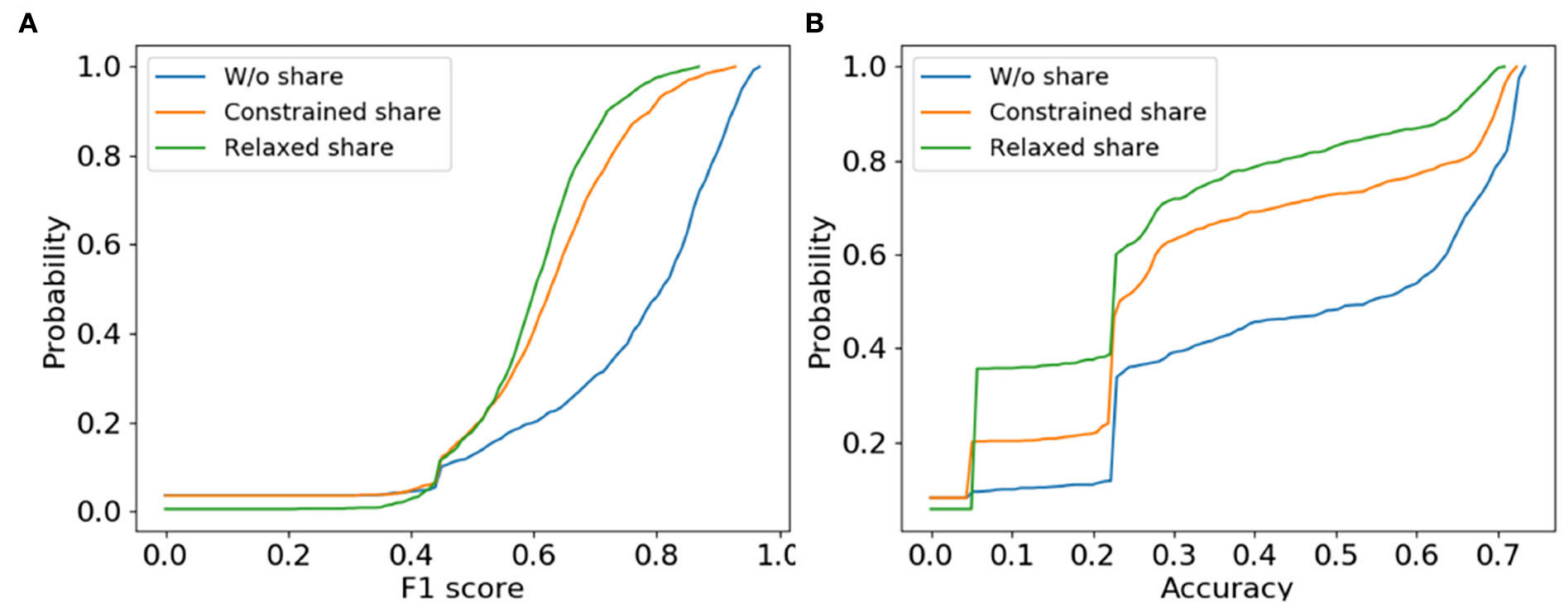
The cumulative distribution of validation accuracies of the 1,000 sampled architectures for AGNN under the constrained/relaxed/without parameter sharing. **(A)** PPI and **(B)** Citeseer.

We study the effectiveness of the proposed constrained parameter sharing to answer question **Q5**. The effective transferred weights should couple into the new architectures to estimate them as accurate as training from scratch. Over AGNN framework, we compare the constrained sharing with the relaxed one in GraphNAS as well as training from scratch. The cumulative distribution of classification performances are shown in Figure 4 for the 1,000 sampled architectures, where we make the observation.

For the cumulative distribution, *X*-axis denotes the cumulative performance and the *Y*-axis is the corresponding probability, where the lower curve has the better estimation of the sampled architectures. ❻ *The constrained parameter sharing strategy estimates the new architectures similar to the ground truth of training from scratch*. Compared with the relaxed sharing, the cumulative distribution curves of the constrained sharing are much closer to those of training from scratch. Given a certain cumulative probability in *Y*-axis, the constrained sharing reaches those neural architectures with better model performances than the relaxed one. That is because the parameter sharing is allowed only among the homogeneous architectures with similar output statistics. Combined with a few epochs to warm up weights in the connection layers, these constraints ensure the shared weights to be effective in the newly sampled architectures.

#### 6.4.6. Influence of architecture modification

**Figure 5:**
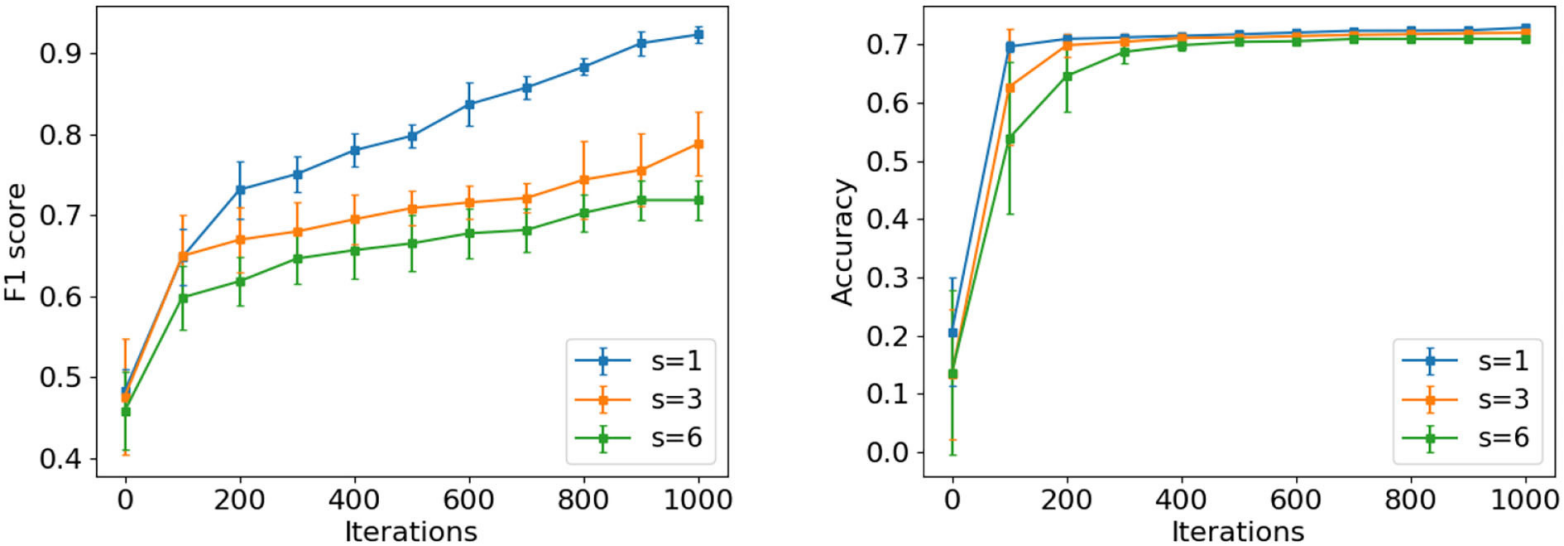
The progression of top-10 averaged performance for AGNN under modification size *s* = 1, 3, and 6 on PPI **(left)** and Citeseer **(right)**.

We study how the different scales of architecture modifications affect the search efficiency of AGNN. While the preserved architecture is modified at the minimum level when modification size *s* = 1, the architecture string will be resampled completely similar to the traditional controller if *s* = 6. Specially, considering *s* = 1, 3, and 6, we show the top-10 architecture progressions on PPI and Citeseer in Figure 5. Since the modification scales affect the coupling of shared weights to the child architectures, AGNN is evaluated under the parameter sharing.

❼ *It is observed that the progression efficiency decreases with the increment of* *s*. Specifically, we re-evaluate the best architectures identified with different modification sizes on PPI. The F1 scores of *s* = 1, 3 and 6 are 0.991, 0.987, and 0.982, respectively, which also decrease with *s*. The benefits of smaller *s* in searching neural architectures are due to the following two facts. First, the offspring tends to have the similar structure and output statistics as the preserved input architecture, since it is generated with a slight modifications. The shared weights would be more effective in the offspring to estimate the model performance accurately. Second, compared with *s* > 1, the controller of *s* = 1 changes the specific module class to obtain the corresponding performance variation. By removing the interference from other module classes, the specific RNN encoder exactly learns the relationship between the module samples of certain class and model performance. Through training the RNN encoder iteratively, it tends to sample good modules to formulate the well-performing architectures in the future search.

#### 6.4.7. Trade-off between performance and time cost

Training from scratch learns the weights of the sampled architectures accurately to improve search reliance, which help approximate to the optimal architecture. However, it incurs an enormous computational cost. Our constrained sharing provides a trade-off between the search performance and computation time. Through transferring weights to avoid complete training, the constrained strategy uses another few epochs to warm up the weights of sampled architectures to promise an accurate model estimation to some degree. The more epochs it takes, the better search performance it may achieve. We study this trade-off by considering the series of warm-up epochs {5, 20, 50} / {1, 5, 10} for the transductive/inductive settings. The discovered model performance and time cost are shown in Table 5. Note that time cost is estimated under the following environments: PyTorch implementation, test machine of GeForce GTX-1080 Ti with 12GB GPU.

**Table 5:** The trade-off between node classification performance and computation time cost for AGNN.

**Model**	**#Epochs**	**Cora**		**Citeseer**		**Pubmed**		**PPI**	
	**T/I**	**Time**	**Accuracy**	**Time**	**Accuracy**	**Time**	**Accuracy**	**Time**	**F1 score**
AGNN-w/o share	200 / 20	27.76	83.6 ± 0.3%	23.42	73.8 ± 0.7%	28.85	79.7 ± 0.4%	34.36	0.992 ± 0.001
AGNN-with share	5 / 1	1.57	41.9 ± 2.5%	4.60	67.7 ± 1.3%	2.51	77.4 ± 0.7%	3.48	0.953 ± 0.055
AGNN-with share	20 / 5	4.34	82.7 ± 0.6%	6.61	72.7 ± 0.7%	7.42	79.0 ± 0.5%	12.57	0.991 ± 0.001
AGNN-with share	50 / 10	11.70	80.2 ± 0.6%	11.09	72.5 ± 0.2%	10.42	79.1 ± 0.2%	22.62	0.991 ± 0.001

Time in second is evaluated to measure the time cost taken to search and train a new architecture. Symbols T and I denote the transductive and inductive learning, respectively. #Epochs *a*/*b* denotes the number of training epochs *a* and *b* for these two learning.

Some notable features about the trade-off could be summarized from Table 5. First, the warm-up epoch of 5/1 is insufficient to train the new architecture well to estimate its classification ability accurately. A worse neural architecture may be identified and tested finally in the given task. Especially in dataset Cora, the classification accuracy is even much worse than the human-invented neural networks. Second, the warm-up epoch of 20/5 achieves a good balance between the search performance and the time cost. It obtains the accuracy or F1 score comparable to training from scratch with 200 epochs, but only consumes a small amount of time. Third, the model performances are similar for the warm-up epochs of 50/10 and 20/5, although the former one takes much more time to train the new architectures. That is because the parameter sharing helps transfer the well-trained weights from the ancestor architecture. A few epochs would be enough to adapt these weights to the new architecture to estimate its classification performance.

#### 6.4.8. Model transfer

Following the model evaluation of NAS in other domains (Pham et al., 2018; Zoph et al., 2018), we delve into the research problem of understanding whether the discovered GNNs could be generalized to the different tasks. To be specific, we transfer the architecture identified on Cora to the node classification tasks on Citeseer and Pubmed. We apply the neural architecture found without parameter sharing by AGNN. Table 6 shows the classification accuracies of the transferred architecture, compared with the original optimal ones in Table 1.

**Table 6:** Test performance comparison of the transferred architecture to the optimal ones on Citeseer and Pubmed.

**Model**	**Citeseer**	**Pubmed**
AGNN-w/o share	73.8 ± 0.7%	79.7 ± 0.4%
Transferred model	71.8 ± 0.7%	78.5 ± 0.4%

❽ *It is observed that the test accuracies of the transferred architecture are a little bit worse than those of the discovered optimal ones*. The experimental results validate our previous research motivations—there is none a graph neural networks could generalize to a series of different graph-structured data. Given a specific graph analysis task, it is crucial to carefully identify a well-performing architecture to optimize the desired performance.

## 7. Conclusion

In this paper, we present AGNN to find the optimal graph neural architecture given a node classification task. The comprehensive search space, RCNAS controller and constrained parameter sharing strategy together are designed specifically for the optimization of elementary, deep, and scalable GNNs. The experimental results show that the discovered neural architectures achieve quite the competitive performances on popular benchmark datasets and large-scale graphs. The proposed RCNAS controller searches the well-performing architectures more efficiently, and the shared weights could be more effective in the offspring network under constraints.

## Data availability statement

The original contributions presented in the study are included in the article/Supplementary material, further inquiries can be directed to the corresponding author.

## Author contributions

KZ and XHu contributed to the whole framework. XHua, QS, and RC contributed to the paper revising. All authors contributed to the article and approved the submitted version.

## Funding

This work partially supported by NSF (#IIS-1750074, #IIS-1900990, and #IIS-18490850).

## Conflict of interest

Author QS was employed by LinkedIn. Author RC was employed by Samsung Research America. The remaining authors declare that the research was conducted in the absence of any commercial or financial relationships that could be construed as a potential conflict of interest.

## Publisher's note

All claims expressed in this article are solely those of the authors and do not necessarily represent those of their affiliated organizations, or those of the publisher, the editors and the reviewers. Any product that may be evaluated in this article, or claim that may be made by its manufacturer, is not guaranteed or endorsed by the publisher.

## Author disclaimer

The views, opinions, and/or findings contained in this paper are those of the authors and should not be interpreted as representing any funding agencies.
