# Supplementary material for: Auto-GNN: Neural architecture search of graph neural networks
Source: Front Big Data. 2022 Nov 17;5:1029307. doi: 10.3389/fdata.2022.1029307 (PMC9714572; doi:10.3389/fdata.2022.1029307)
Supplement: Supplementary file 1 [file Data_Sheet_1.PDF]

Supplementary Material

1 DATASETS

We utilize Cora, Citeseer and Pubmed Sen et al. (2008) for the transductive learning, and use PPI for the inductive learning Zitnik and Leskovec (2017). The large-scale graphs are Reddit Hamilton et al. (2017) and ogbn-products Hu et al. (2020). These benchmark datasets are commonly used for studying node classification task, and the data statistics is given in Table S1.

Table S1. Dataset statistics, where T and I denote the transductive and inductive learning, respectively.

	Cora	Citeseer	Pubmed	PPI	Reddit	ogbn-products
Setting	T	T	T	I	T	T
#Nodes	2708	3327	19717	56944	232,965	2,449,029
#Features	1433	3703	500	50	602	100
#Classes	7	6	3	121	47	41
#Training Nodes	140	120	60	44906 (20 graphs)	66%	78%
#Validation Nodes	500	500	500	6514 (2 graphs)	10%	2%
#Testing Nodes	1000	1000	1000	5524 (2 graphs)	24%	90%s

2 SEARCH SPACE DETAILS

The search space includes eight dimensions of modules. A wide variety of candidate options are provided for each dimension to support the neural architecture search. In detail, the hidden dimension is explored within set $\{4,\ 8,\ 16,\ 32,\ 64,\ 128,\ 256\}$. The attention function is selected from set $\{\mathrm{CONSTANT},\ \mathrm{GCN},\ \mathrm{SYMGAT},\ \mathrm{COS},\ \mathrm{LINEAR},\ \mathrm{GERELINEAR}\}$. We list their mathematical expressions in Table S2 Gao et al. (2019). The aggregate function contains $\{\mathrm{SUM}, \mathrm{MEAN}, \mathrm{MAX}\}$. The attention head contains the following candidates: $\{1,\ 2,\ 4,\ 6,\ 8,\ 16\}$. The combine function is provided by set $\{\mathrm{IDENTITY},\ \mathrm{MLP}\}$. MLP is a 2-layer perceptron with a fixed hidden dimension of 128. The activation function includes the series $\{\mathrm{Sigmoid}, \mathrm{Tanh}, \mathrm{ReLU}, \mathrm{Linear},\ \mathrm{Softplus},\ \mathrm{LeakyReLU},\ \mathrm{ReLU6},\ \mathrm{ELU}\}$. The skip connections is to choose all the previous layers. The batch size is sampled from $\{128, 256, 512, 1024, 2048\}$.

3 TRAINING DETAILS

Following the model configurations in baselines Velickovic et al. (2017); Gao et al. (2018), we search the two-layer and three-layer GNN architectures for the transductive and inductive learning, respectively. We train the shared weights in the sampled neural architectures by utilizing the training set, and update the controller via receiving reward from the validation set. To be specific, we introduce the training details of transductive learning, inductive learning and controller as follows.

3.1 Transductive Learning.

Herein we explore a two-layer GNN architecture in the predefined search space. Except that the neural architecture is updated iteratively during the search progress in NAS methods, we have the same training configuration to those in the human-invented baselines. To deal with the issue of small training set, we

Table S2. The set of attention functions, where symbol $||$ denotes the concatenation operation, $\vec{a}$, $\vec{a}_l$ and $\vec{a}_r$ denote the trainable vectors, and W_G denotes the trainable matrix.

Attention Mechanisms	Equations
CONSTANT	1
GCN	$\frac{1}{\sqrt{\|\mathcal{N}(i)\|\|\mathcal{N}(j)\|}}$
GAT	$\mathrm{LeakyReLU}(\vec{a}(W^{(k)}x_i^{(k-1)}\|\|W^{(k)}x_j^{(k-1)}))$
SYM-GAT	$a_{ij}^{(k)} + a_{ji}^{(k)}$ based on GAT
COS	$\vec{a}(W^{(k)}x_i^{(k-1)}\|\|W^{(k)}x_j^{(k-1)})$
LINEAR	$\tanh(\vec{a_l}W^{(k)}x_i^{(k-1)} + \vec{a_r}W^{(k)}x_i^{(k-1)})$
GERE-LINEAR	$W_G\tanh(W^{(k)}x_i^{(k-1)} + W^{(k)}x_i^{(k-1)})$

apply L2 regularization with loss scale $\lambda = 0.0005$. Dropout rate of 0.6 is applied to both teh layers' inputs as well as the attention coefficients during training. For Pubmed dataset, L2 regularization is strengthened to $\lambda = 0.001$.

For each sampled architecture, weights are initialized using Glorot initialization Glorot and Bengio (2010) and trained with Adam optimizer Kingma and Ba (2014) to minimize the cross-entropy loss. We set the initial learning rate of 0.01 for Pubmed and 0.005 for Cora and Citeseer. We have two different settings to train a new offspring architecture: with parameter sharing and without weight sharing. The former one has a small warm-up epochs of 20, while the later one has 200 training epochs.

3.2 Inductive Learning.

We explore a three-layer GNN architecture. The skip connection between the intermediate graph convolutional layers is included to improve the representation learning. Since dataset PPI is sufficiently large for training, the L2 regularization and random dropout are removed from GNN model. The batch size of 2 graphs is employed during training.

We have the same parameter initialization and optimizer as the transductive learning. The initial learning rate is set to 0.005. The warm-up epoch number is 5 under the setting with parameter sharing, and it is 20 under the setting without parameter sharing.

3.3 Controller.

For each module class, RNN encoder is realized by an one-layer LSTM with 100 hidden units. Weights are initialized uniformly in $[-0.1, 0.1]$, and trained with Adam optimizer at a learning rate of 3.5×10^{-4}. Following the controller configurations in the previous NAS work, we use a $\tanh$ constant of 2.5 and a sample temperature of 5.0 to the hidden output. Total of 1000 architectures are explored iteratively during the search progress, and evaluated to obtain reward for updating controller. Reward to the policy gradient is given by the following combination: validation performance and controller entropy weighted by 1.0×10^{-4}.

4 BEST ARCHITECTURE EXPLANATION

We illustrate and explain the best architecture discovered by AGNN, expecting to shed light on the future model design. The neural architecture of GNN is given by a string of length $6n$, where n denotes the number of graph convolutional layers. Considering datasets PPI and Pubemd, we show the their architectures in

Table S3. The best architecture discovered on PPI by AGNN.

Modules	Layer 1	Layer 2	Layer 3
Hidden dimension	64	64	121
Attention function	CONSTANT	CONSTANT	CONSTANT
Attention head	16	16	16
Aggregate function	SUMMATION	MEAN	MEAN
Combine function	IDENTITY	MLP	MLP
Activation function	ReLU6	ReLU	Linear

Table S4. The best architecture discovered on Pubmed by AGNN.

Modules	Layer 1	Layer 2
Hidden dimension	4	3
Attention function	COS	GERE-LINEAR
Attention head	16	6
Aggregate function	SUMMATION	SUMMATION
Combine function	IDENTITY	IDENTITY
Activation function	ELU	ELU

Tables S3 and S4, respectively, in order to compare the architecture difference between the transductive and the inductive learning.

Some notable features could be concluded from the identified architecture in Table S3 for PPI. First, the attention functions in all of the three layers are realized by $\mathrm{CONSTANT}$, which means that the importance coefficients $a_{i,j}^{(k)}$ of all neighbors are given by 1. As shown in Equation (**??**), the graph convolutions will aggregate the neighbors without preference. Note that PPI dataset consists of a series of 24 graphs, where the trained neural networks will be transferred and evaluated on the independent testing graphs. Since the adjacency structures of the training and testing graphs may be different, the learned attention function is not adaptive to the new graph. On the contrary, the attention function of CONSTANT avoids introducing the prior knowledge to the new testing environment when aggregating the neighborhoods. Second, the aggregate functions of $\mathrm{SUMMATION}$ or MEAN are more preferable than $\mathrm{MAXPOOLING}$, and the combine function of MLP is applied to improve the representation learning. This is consistent with the theoretical analysis in work Xu et al. (2018), which ranks the expression powers of different aggregation and combination functions. Being different with the transductive learning on Cora, the mean aggregation instead of max pooling is made use here. One of the possible reasons is that the mean aggregation measures the average neighbor distribution Xu et al. (2018), which may be more easier to generalize to the independent testing graphs. Third, a large numbers of the hidden dimension and attention head are applied to provide more trainable weights, which will improve the model learning ability in the complex inductive scenario.

Compared with the one on PPI, there are some different features on the identified architecture in Table S4 for Pubmed. First, attention functions of COS and $\mathrm{GERE\text{-}LINEAR}$ are found to assign the various importance to the different neighbors, instead of using the $\mathrm{CONSTANT}$. Note that Pubmed dataset is evaluated under transductive setting. The well-trained neural network is tested on the nodes that belong to the same graph with the training set. The trainable attention functions aggregate information from the most important neighbors to improve the node representation learning. Second, only $\mathrm{IDENTITY}$ function is selected to combine the representations of node itself and its neighbors, which is contradictory to Corollary 6 in Xu et al. (2018). That is because the node number in Pubmed is smaller than that in PPI, which means that the transductive learning task of Pubmed is much easier. For such a simple task, function $\mathrm{IDENTITY}$ may be powerful enough to learn the node representations, at the same time avoiding the over-fitting

issue. Third, a small hidden dimension and attention head are applied. The node input features in Pubmed are given by bag-of-words, which are a sparse vector. The smaller dimension helps compress the crucial information and learns the abstract embedding.

5 DEEP ARCHITECTURE ILLUSTRATION

We list the skip connection indexes of 16-layer GNN searched on Cora, At layer k, the skip connections are given by a vector of length k, where index i ($i = 0, \cdots, k-1$) indicates whether the intermediate layer i is connected to current layer or not. The skip connection indexes of 16-layer GNN searched on Cora are as follows, where the skip connections are frequently selected to relieve the over-smoothing.

Layer 1: [0];

Layer 2: [0, 1];

Layer 3: [1, 1, 1];

Layer 4: [0, 1, 0, 1];

Layer 5: [1, 0, 1, 0, 1];

Layer 6: [0, 1, 1, 0, 0, 0];

Layer 7: [1, 1, 1, 1, 1, 1, 0];

Layer 8: [1, 1, 1, 1, 1, 0, 1, 1];

Layer 9: [0, 1, 1, 0, 1, 1, 1, 0, 1];

Layer 10: [0, 0, 1, 1, 1, 0, 1, 1, 1, 0];

Layer 11: [1, 0, 0, 1, 1, 0, 1, 0, 0, 0, 0];

Layer 12: [0, 0, 0, 0, 1, 1, 1, 1, 0, 1, 1, 0];

Layer 13: [1, 1, 0, 1, 0, 1, 1, 1, 0, 0, 0, 0, 1];

Layer 14: [0, 0, 0, 0, 1, 1, 1, 0, 0, 0, 1, 1, 1, 0];

Layer 15: [0, 0, 0, 1, 0, 0, 1, 1, 1, 0, 0, 1, 0, 0, 1];

Layer 16: [1, 0, 0, 0, 0, 0, 1, 1, 0, 1, 1, 0, 1, 0, 0, 0].

6 SCALABLE MODEL ILLUSTRATION

We list the scalable architecture searched on Reddit. Please note that we optimize the module choices of one layer, which is repeated to the 3-layer GNNs. To be specific, the module choices of one layer are: [Skip connection to the first layer; Attention layer: GAT; Attention head: 4; Hidden units: 256; Batch size: 2048; Activation function: ReLU].

7 ENVIRONMENTS

All the NAS approaches are implemented with PyTorch, and tested on the machine with a GeForce GTX-1080 Ti 12 GB GPU. The maximum of 0.5 GPU day is used for search.

REFERENCES

Gao, H., Wang, Z., and Ji, S. (2018). Large-scale learnable graph convolutional networks. In *SIGKDD* (ACM), 1416–1424

Gao, Y., Yang, H., Zhang, P., Zhou, C., and Hu, Y. (2019). Graphnas: Graph neural architecture search with reinforcement learning. *arXiv*

Glorot, X. and Bengio, Y. (2010). Understanding the difficulty of training deep feedforward neural networks. In *AISTATS*. 249–256

Hamilton, W., Ying, Z., and Leskovec, J. (2017). Inductive representation learning on large graphs. In *NeuIPS*. 1024–1034

Hu, W., Fey, M., Zitnik, M., Dong, Y., Ren, H., Liu, B., et al. (2020). Open graph benchmark: Datasets for machine learning on graphs. *arXiv preprint arXiv:2005.00687*

Kingma, D. P. and Ba, J. (2014). Adam: A method for stochastic optimization. *arXiv*

Sen, P., Namata, G., Bilgic, M., Getoor, L., Galligher, B., and Eliassi-Rad, T. (2008). Collective classification in network data. *AI magazine*

Velickovic, P., Cucurull, G., Casanova, A., Romero, A., Lio, P., and Bengio, Y. (2017). Graph attention networks. *arXiv* 1

Xu, K., Hu, W., Leskovec, J., and Jegelka, S. (2018). How powerful are graph neural networks? *CoRR* abs/1810.00826

Zitnik, M. and Leskovec, J. (2017). Predicting multicellular function through multi-layer tissue networks. *Bioinformatics* 33, i190–i198
